# Association between e-cigarette use and myocardial infarction: a systematic review and meta-analysis

**DOI:** 10.1186/s43044-023-00426-6

**Published:** 2023-11-30

**Authors:** Muhammad Talal Ashraf, Asim Shaikh, Muhammad Khuzzaim Shakeel Khan, Naseer Uddin, Muhammad Arham bin Kashif, Syed Hassan Ahmed Rizvi, Hammad Khalid, Stafford Jude Sam, Affan Sohail

**Affiliations:** 1 Department of Internal Medicine, Dow University of Health Sciences, Karachi, 74200 Pakistan; 2 Department of Medicine, Aga Khan University Hospital, Karachi, 74800 Pakistan; 3 Karachi, Pakistan

**Keywords:** Cardiology, Cardiovascular, Electronic cigarettes, Myocardial infarction, Myocardial ischemia, Vape

## Abstract

**Background:**

The popularity of e-cigarettes has risen dramatically over the last few years, particularly among the younger population. Although the use of combustible cigarettes has established evidence to be associated with the development of several adverse cardiopulmonary diseases, the investigations regarding the prospective long-term effects of e-cigarette use on the cardiovascular system have just begun. We set to investigate if there is an association between the history of MI and e-cigarette use among smokers and non-smokers?

**Methods:**

The current review aims to assess the association of myocardial infarction with e-cigarette consumption. PubMed, Google Scholar, and Cochrane Central Register of Controlled Trials (CENTRAL) were queried up to October 2022 to identify articles assessing the incidence of myocardial infarction among e-cigarette users. Data were meta-analyzed using a random-effects model to derive odds ratios (OR) and 95% confidence intervals.

**Results:**

Nine studies involving 984,764 patients were included. The mean age of e-cigarette smokers was less than the controls, and female participants dominated the sample size. E-cigarette users were associated with increased odds of MI than non-users [OR = 1.44; 95% CI (1.22, 1.74); *P* < 0.0001]. Dual users were also associated with increased odds of MI with large effect when compared to non-users [OR = 4.04; 95% CI (3.40, 4.81); *P* < 0.00001].

**Conclusions:**

Dual use is associated with an increased risk of MI than e-cigarette use only. Similarly, dual and solely e-cigarette consumption patterns of nicotine delivery are at a higher risk of MI than non-smokers.

**Supplementary Information:**

The online version contains supplementary material available at 10.1186/s43044-023-00426-6.

## Background

Electronic cigarettes (e-cigs) are devices that provide a different mechanism of nicotine delivery eliminating the need for combustion of tobacco. They heat solutions containing nicotine, flavoring, additives, propylene glycol, and/or vegetable glycerin. First marketed in 2007, the last 10 years have seen a huge increase in e-cig use among non-smoker populations partly because of their marketing as a safer alternative to traditional tobacco cigarette smoking. An estimated 12.6% of American adults and approximately 48.5 million EU citizens have used e-cigs at least once [1, 2]. Combustible compounds present in tobacco are the main reason behind their harmful effects, and thus, e-cigs are presented as a safer alternative.

However, the composition of e-cigs varies widely among the different brands, and the fluids used. Acetaldehyde, formaldehyde, and acetone are harmful chemicals normally present in tobacco smoke but have also been found in e-cigs, considering that these chemicals are shared between both nicotine delivery methods, e-cigs should perhaps not be marketed as a ‘safer’ form of smoking [3–5].

Nicotine consumption causes the release of catecholamines which leads to hemodynamic effects [6] such as increase in blood pressure and heart rate [7, 8]. E-cigs have also been associated with oxidative stress and endothelial dysfunction [8]. In addition, their use has also been linked to platelet activation which might contribute to accelerated atherosclerosis [9].

In addition, an estimated 17.9 million people died in 2019 due to cardiovascular diseases (CVDs) which accounted for 32% of all global deaths that year. Of these, 85% were due to MI and stroke. Some important modifiable risk factors for CVD include an unhealthy diet, tobacco use, lack of physical activity, and alcohol consumption [10]. Tobacco smoking is a strong risk factor for acute MI [11].

While the literature mentioned above describes the adverse effects of e-cig use on cardiovascular outcomes, our knowledge in this domain is still far from complete, especially regarding the occurrence of MI in e-cig users. To fill this important gap in knowledge, we conducted a comprehensive systematic review and meta-analysis evaluating the association between e-cig consumption and the risk of MI, along with other cardiovascular outcomes.

## Methods

Preferred Reporting Items for Systematic Reviews and Meta-Analysis (PRISMA) guidelines were followed while conducting this systematic review and meta-analysis [12]. We registered our study protocol on PROSPERO, University of York (CRD42022362625). The primary outcome of this study was the diagnosis of MI in e-cig smokers as defined by the included studies.

### Literature search

Electronic search through Medline, Google Scholar (starting 10 pages), EMBASE, Scopus, Cochrane Central Register of Controlled Trials (CENTRAL), and Web of Science was performed from their inception till September 1st, 2022, with the search string: (vape OR electronic nicotine delivery system* OR e-cig* OR electronic cigarette*) AND (myocardial infarct* OR coronary heart disease OR acute coronary syndrome OR angina OR ischemic heart disease OR cardiovascular disease). A previously published search string was also incorporated [13]. We also searched the included studies’ references, related articles, and suggested results from the PubMed database for studies that fulfilled our inclusion criteria. Gray literature was also incorporated in our search.

### Data screening and eligibility

The search results were exported to EndNote, Version 20 (Clarivate Analytics, Philadelphia, USA) for the removal of duplicate studies. Then, the remaining results were moved to an Excel (v2019, Microsoft, Redmond, Washington, USA) spreadsheet with general details (title, authors, abstract, year of publication, journal). Screening of titles and abstracts for potential studies was done by 2 independent authors (AS and MTA) and a 3rd author (AS) was involved to resolve disagreements and discrepancies. The inclusion criteria for study selection were the following; (1) human only study that was either an investigational (randomized or non-randomized controlled trial) or an observational study (cross-sectional, prospective, retrospective, case–control or post hoc analysis of a cohort data) or research letters or conference abstracts containing information fulfilling our inclusion criteria, (2) no restriction on the follow-up duration and age group of participants, gender/race of the participant, country, language of the article, was followed, (3) study with a minimum of 10 participants were selected, (4) study that reported risk ratios, odds ratios, episodes of MI were included. Exclusion criteria were, (1) studies not reporting MI events, (2) books, reviews, case reports, thesis, duplicated studies or incomplete data, animal studies, and previous systematic reviews/meta-analyses.

### Data extraction and quality assessment

Data extraction was performed by two independent authors (NU and MKSK), the extraction sheets were cross-matched, and disparities were resolved with the consensus of a third author (AS). A data extraction sheet in excel was created including the study characteristics (title, author, year of publication, sample size of the study), participants’ characteristics (age, sex, any previous history of diabetes, and hypertension race and BMI) and outcomes (events of MI in various groups of e-cig users and non-users, odds ratios, risk ratios). E-cig use compared to non-users was established, which was further divided into daily (everyday users) and someday users. Everyday users were daily consumers. Someday users were defined as those not consuming e-cigs daily but at least 2 days a week, and smokers using both combustible cigarettes and e-cigs (dual users) were compared to non-users. We also analyzed former e-cig users, participants who answered no to currently consuming any e-cigs at the time of the survey but have used them in their life, to see if there was a prolonged effect. Anytime use, defined as e-cig consumption for at least 10 days in their life, was collected as well. Non-users were defined to be non-active consumers of e-cigs. The quality assessment of these studies included was assessed by 2 independent authors (AK and SHAR) using the Axis Tool, which is a scale used to assess the quality of cross-sectional studies for systematic reviews and meta-analyses [14].

### Statistical analysis

Review Manager (Version 5.3, Copenhagen: The Nordic Cochrane Centre, The Cochrane Collaboration, 2014) was used for the statistical analyses. Dichotomous data were pooled as odds ratio (OR) with 95% confidence intervals (CIs) using the Mantel–Haenszel method. To depict the outcome of the analyses, forest plots were obtained and reported. Adjusted effect sizes were pooled through the inverse variance weighted method using a random-effects model. The Higgins I^2^ statistic was looked at to assess heterogeneity, and an *I*^2^ value of > 50% was considered to indicate significant heterogeneity. We omitted one study at a time to assess whether the results may be disproportionately influenced by any single study and to check for sensitivity analysis across all the outcomes. A *p* value of < 0.05 was considered significant in all cases.

The primary outcomes were the incidence of MI in users of e-cigs and dual users compared to non-users. The secondary outcomes were the incidence of MI in everyday, someday, and former users of e-cigs compared to non-users. Another secondary outcome was to compare anytime users versus those who have never used e-cigs.

## Results

**Fig. 1:**
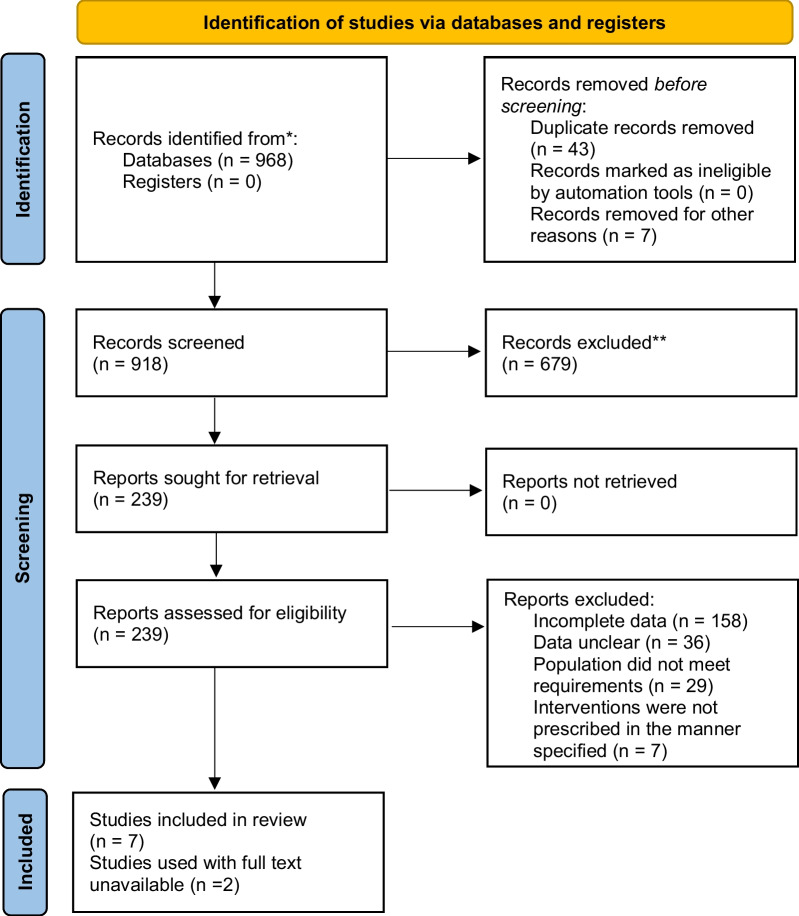
PRISMA flowchart

Data of 984,764 participants were acquired from nine studies. The literature search was conducted under the PRISMA guidelines and the reasons for exclusion are depicted in the PRISMA flowchart (Fig. 1). The original results were 968 and were narrowed down to 918 after duplicates, and letters were removed. The full text screening yielded nine studies. All selected studies were cross-sectional analyses of different surveys conducted in the US [15–23]. The quality assessment tables were acquired to measure the characteristics of included studies. Seven out of nine studies included in our analysis were of high quality as they fulfilled most of the assessment criteria while the remaining 2 were graded poor owing to the unavailability of full texts (Additional file 1).

### Baseline characteristics

The standard characteristics of the population pool across the selected studies are shown in Table 1. Female respondents dominated the respondent pool in most of the studies. The mean age for e-cig consumers was lower in all surveys. The ethnicity forming the bulk of the pool, as reported by two surveys, was Caucasian, followed by Hispanic, African American, and then Asian. The mean BMI was similar across all participants.

**Table 1:** Table of baseline characteristics

Study	Database used	Sample size	Gender		Age Mean (SD)	
			Male N (%)	Female N (%)	E-cigarette	Non-E-cigarette
Farsalinos [15]	NHIS 2016–17	59,579	26,984 (45.3)	32,595 (54.7)	42.1	50.8
Critcher [16]	NHIS 2014, 2016–19	175,546	80,742 (46.0)	94,804 (54.0)		
Vindhyal [17]	NHIS 2014, 2016–17	96,467			32.9	40.4
Alzahrani [18]	NHIS 2014, 2016	69,046	30,837 (44.7)	38,209 (55.3)	42.4 (8.33)	51.6 (18.48)
Ndunda [19]	BRFSS 2016	410,651	211,485 (51.5)	199,166(48.5)	44	57
Falk [20]	NHIS 2014, 2016–18	84,553				
Wang [21]	HHS 2013–17	39,747	12,047 (30.3)	27,600 (69.4)		
Vindhyal [22]	NHIS 2014, 2016–18	16,855	10,669 (63.3)	6,156 (36.5)	26.7	44
Rodu [23]	Path 1 wave	32,320	15,546 (48.1)	16,774 (51.9)		

### Primary outcomes

#### Users vs non-users

**Fig. 2:**
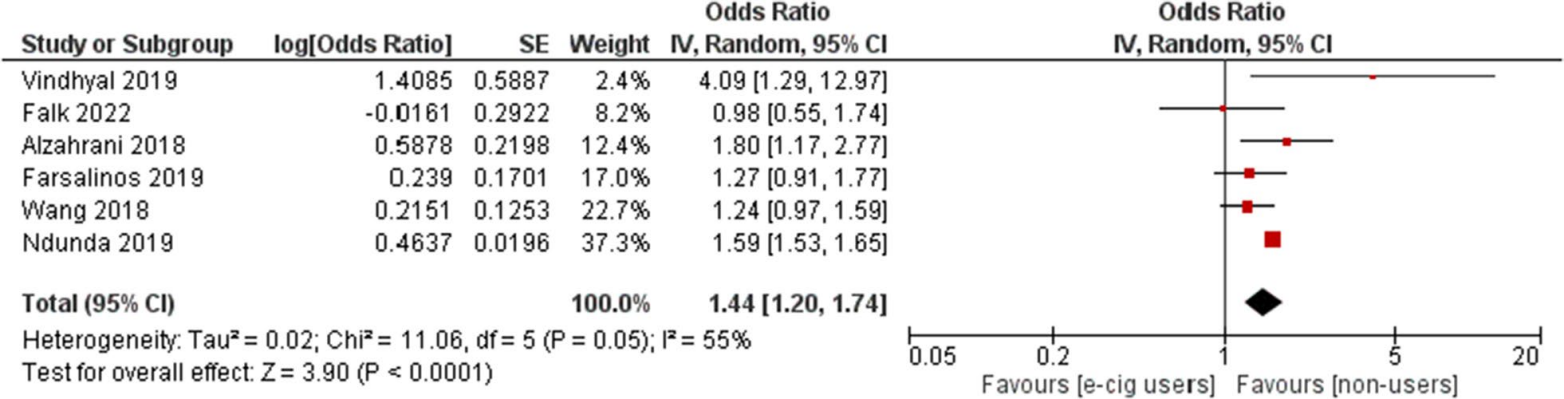
E-cigarette users vs non-users

Users of e-cigs were found out to be associated with increased odds of MI [OR = 1.44; 95% CI (1.22, 1.74); *P* < 0.0001] (Fig. 2). In users and non-users, removing Ndunda et al. [20] and Vindhyal et al. [18] reduced heterogeneity to 8% from 55%, primarily because these studies were conference abstracts.

#### Dual users vs non-users

**Fig. 3:**
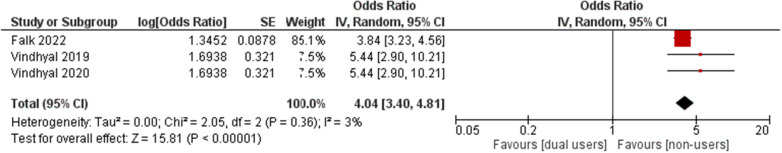
Dual users vs non-users

There was a large, 304%, increase in the occurrence of MI in dual users [OR = 4.04; 95% CI (3.40, 4.81); *P* < 0.00001] (Fig. 3).

### Secondary outcomes

#### E-cigs everyday vs non-users

**Fig. 4:**
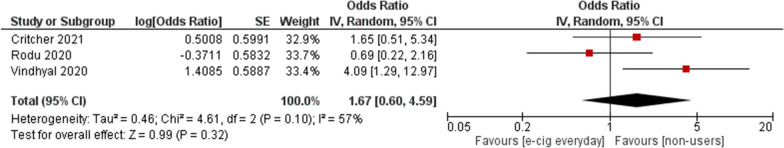
E-cigarettes everyday vs non-users

The findings in this subgroup are calculated to distinguish if everyday consumption carries the greater weight of the earlier comparison. An insignificant association was established with MI history [OR = 1.67; 95% CI (0.60, 4.59); *P* = 0.32] (Fig. 4). For everyday and non-users, the value was reduced significantly by deselecting either Vindhyal (7%) et al. or Rodu et al. (14%)[23], from 57%, however, only deleting Rodu helped in switching the OR to be significant [OR = 2.62; 95% CI (1.07, 6.37); P = 0.03].

#### E-cigs somedays vs non-users

**Fig. 5:**
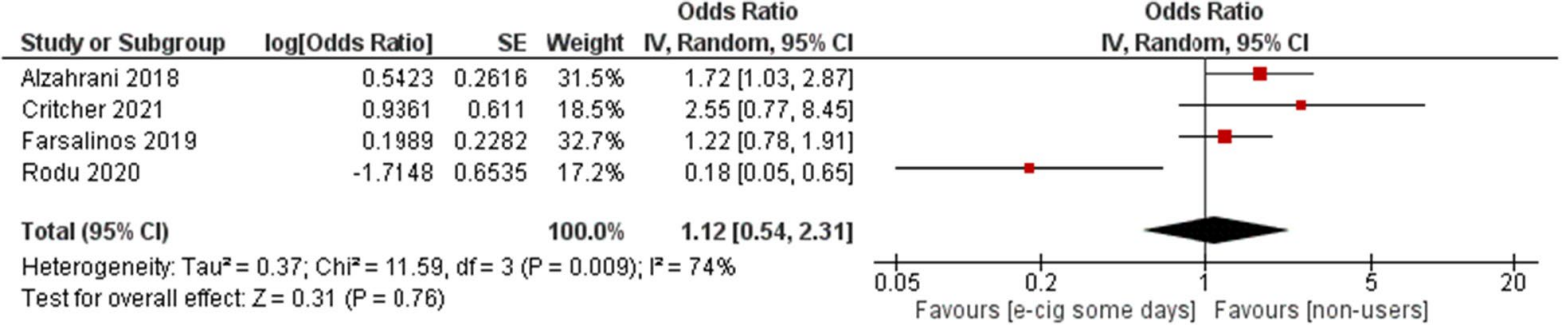
E-cigarettes Somedays vs non-users

Those who occasionally took up e-cigs occasionally were considered for this subgroup. There was an insignificant relationship between occasional use and MI [OR = 1.12; 95% CI (0.54, 2.31); *P* = 0.76] (Fig. 5). Among someday users, the only study in the funnel plot with any significant deviation was Rodu et al. (Additional file 1: Fig. S1). Once Rodu et al. [23] are eliminated from somedays vs non-user, the heterogeneity approaches zero while also rendering the results significant [OR = 1.48; 95% CI (1.07, 2.04); *P* = 0.02].

#### E-cigs former vs non-users

**Fig. 6:**
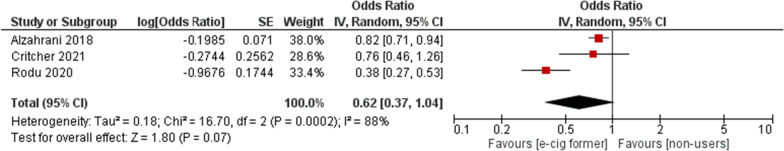
E-cigarettes former vs non-users

This comparison was undertaken to understand if the use of e-cigs, prior to the conductance of these surveys, displayed any relevance. An insignificant association was observed [OR = 0.62; 95% CI (0.37, 1.04); *P* = 0.07] (Fig. 6). Side-lining Rodu et al.[23] for the former users’ analysis, the heterogeneity value turns zero from 88%, while also turning the result significant [OR = 0.82; 95% CI (0.71, 0.93); *P* = 0.003].

#### E-cigs anytime use vs never users

**Fig. 7:**
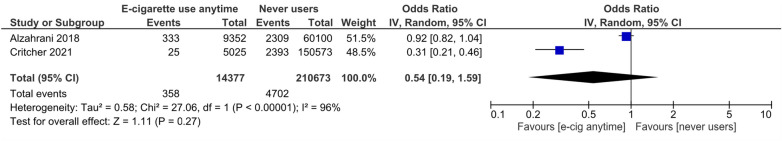
E-cigarettes anytime users vs never users

The use of e-cigs at some point in the participants’ life for a significant duration was evaluated to check if e-cig ingredients may leave an effect and have some association with the occurrence of MI. This category involved everyday, someday and former consumers. No significant association was established between the exposure and outcome [OR = 0.54; 95% CI (0.18, 1.62); *P* = 0.27] (Fig. 7).

## Discussion

For our meta-analysis, we pooled results from nine different cross-sectional analyses based on surveys. We compared e-cig users, dual users including those that also consumed combustible cigarettes and non-users to find out which group(s) experienced an increased association of MI. Our study deduced that e-cig users including users that smoked everyday as well as those that smoked occasionally had a higher incidence of MI as compared to non-users. Dual users, however, appeared to have a substantial (304%) increase in the occurrence of MI as compared to non-users.

Our primary outcome showed that the incidence of MI was higher in individuals that smoked e-cigs currently as compared to non-users. This outcome is supported by another published meta-analysis by Sharma et al. [13] which also concluded that the risk of MI was higher in e-cig users as compared to non-users, however, the risk imposed by e-cigs was half of that of the risk caused by smoking traditional combustible cigarettes. This could be attributed to the fact that nicotine is used in e-cigs [24] and that has traditionally been established to be a major risk factor for MI in people who have consumed combustible tobacco smokes or e-cigs [25, 26].

In addition, our study yielded no significant association between the incidence of MI and different e-cig usage frequencies which included non-users, occasional users, and former users. The incidence of MI in everyday e-cig users vs non-users was not significantly associated, which could possibly point toward the indication that the greater the consumption of e-cigs, the more likely it is to be involved in a MI event, due to the toxic chemicals in the vaping liquid being consumed at a much larger frequency. Similar insignificant results were obtained when the occurrence of MI was evaluated in people who consumed e-cigs occasionally versus non-users and in people who formerly used e-cigs versus non-users. This could be theorized to be due to the major organs recovering from the chemicals that might have accumulated or caused disruption, and now the organs have regenerated healthier tissues. An additional category of all time e-cig users which included everyday users, former users and occasional users was compared with non-users to evaluate for an increased risk for MI; the results for this comparison were also insignificant. Former users could have potentially skewed the analysis and as already seen; occasional users were not found to be at significant risk.

The link between e-cigs and an increased risk of MI is further supplemented by recent evidence which has showed that e-cigs have a contribution in causing significant mortality and morbidity, especially through the generation of oxidative stress which can lead to atherosclerosis [10]. In addition, e-cig use has been directly linked with increased platelet aggregation [11]. Furthermore, a National Health Interview Survey cross-sectional analysis revealed e-cigarette usage was linked to an increased risk of MI, circulatory derangements, and stroke [22]. The liquid in an e-cig which is heated to generate the aerosol includes solvent, water, and nicotine [24]. Nicotine together with the solvents like formaldehyde, acrolein and acetaldehyde has been found to have additional jeopardizing health effects [25]. In some animal studies, nicotine has been found to cause an acutely increased activation of platelets, presumably through the release of epinephrine [26]. The smoking of e-cigs has also been linked to poor general systemic health including cardiac inflammation which leads to cardiovascular disease [27].

A few reasons attributing to why the heterogeneity is high in a few analyses could be that not all the studies operated with the same surveys and carried out their analyses in the same manner to each other. Essentially, the methodology of the studies is different and since these were cross-sectionals the method of acquiring data from the original participants was found to be slightly different in a few of the cross-sectional studies. The funnel plot (Additional file 1: Fig. S1) represents Rodu et al. to be the farthest away due to publication bias, which could be as a result of reporting bias since significant findings are more likely to be reported.

The risk appears dose-dependent with everyday use showing a non-significant 67% increase in MI, while former use was not significantly associated. This implies daily exposure has the strongest association. The findings challenge perceptions of e-cigarettes being harmless alternatives and highlight the lack of long-term safety data. Regulation needs to account for potential population health impacts.

Public health messaging should make clear that e-cigarettes substantially increase MI risk, especially with daily use. Claims of safety require re-evaluation. Stricter regulations on e-cigarette manufacturing, labeling, marketing and sales to minors may be indicated based on mounting cardiovascular safety concerns. Patients should be screened for e-cigarette use and counseled about potential CV and other health risks. Discouraging dual use should be a priority. More longitudinal research is required to establish temporal relationships and eligibility criteria for CV outcomes should be standardized across studies.

## Limitations

Our study had some considerable limitations. Firstly, all included studies are cross-sectional analyses. Secondly, former users could not be accurately differentiated based on prior usage as the studies restricted them to those who were not active consumers anymore, with no data on previous usage. This study also could not compare e-cig users to combustible cigarette smokers due to unavailability data. In addition, combustible cigarette smokers could not be eliminated from the data provided by some studies so slight inaccuracy may have been introduced.

This meta-analysis includes the largest sample size and outcome measures as compared to other meta-analyses of its kind. This is the first review to analyze the association of MI with dual users and how double consumption is related to MI. All studies contribute healthy sample sizes maintaining heterogeneity at acceptable levels.

This is a meta-analysis that attempts to identify exposure outcome relationships between e-cig use and MI. It incorporates studies that reported probability of MI with exposure to electronic cigarettes, however, not all of them accounted for time varying use of combustible tobacco which is a dominant risk factor and confounder. At the same time, the quality assessment yields most of these studies to be good quality. Sharma et al. [13] analyze these studies in the same manner, while our review has greater variations and more studies in most comparison. This review also assesses greater degrees of frequency of e-cigarette consumption to allow greater margins to be drawn.

## Conclusions

The risk of MI in e-cig users and dual users is higher as compared to non-users. Simultaneously, those who were formerly accustomed to e-cigs or have used it at some point in their lives did not display increased risk for MI. This represents the phenomenon that the greater the frequency of e-cigarette consumption, the more likely it is to be linked to MI.

### Supplementary Information

**Additional file 1. e-Appendix 1.** AXIS quality assessment tool for observational studies. **Figure 1:** Funnel plot for some-days users vs non-users.

## Data Availability

Available on request.

## Abbreviations

CI: Confidence interval
CVD: Cardiovascular disease
E-cigs: Electronic cigarettes
MI: Myocardial infarction
OR: Odds ratio
RCT: Randomized controlled trial
