# Supplementary material for: Association between e-cigarette use and myocardial infarction: a systematic review and meta-analysis
Source: Egypt Heart J. 2023 Nov 30;75:97. doi: 10.1186/s43044-023-00426-6 (PMC10689622; doi:10.1186/s43044-023-00426-6)

**Supplementary**

**e-Appendix 1**

**AXIS quality assessment tool for observational studies:**

1. ***Konstantinos et al 2019***

|  | **Question** | **Yes** | **No** | **Don’t know/ Comment** |
| --- | --- | --- | --- | --- |
| ***Introduction*** | | | | |
| 1 | Were the aims/objectives of the study clear? | ● |  |  |
| ***Methods*** | | | | |
| 2 | Was the study design appropriate for the stated aim(s)? | ● |  |  |
| 3 | Was the sample size justified? |  | ● |  |
| 4 | Was the target/reference population clearly defined? (Is it clear who the research was about?) | ● |  |  |
| 5 | Was the sample frame taken from an appropriate population base so that it closely represented the target/reference population under investigation? | ● |  |  |
| 6 | Was the selection process likely to select subjects/participants that were representative of the target/reference population under investigation? | ● |  |  |
| 7 | Were measures undertaken to address and categorize non-responders? |  | ● |  |
| 8 | Were the risk factor and outcome variables measured appropriate to the aims of the study? | ● |  |  |
| 9 | Were the risk factor and outcome variables measured correctly using instruments/measurements that had been trialed, piloted or published previously? | ● |  |  |
| 10 | Is it clear what was used to determined statistical significance and/or precision estimates? (e.g. p-values, confidence intervals) | ● |  |  |
| 11 | Were the methods (including statistical methods) sufficiently described to enable them to be repeated? | ● |  |  |
| ***Results*** | | | | |
| 12 | Were the basic data adequately described? | ● |  |  |
| 13 | Does the response rate raise concerns about non-response bias? |  | ● |  |
| 14 | If appropriate, was information about non-responders described? |  | ● |  |
| 15 | Were the results internally consistent? | ● |  |  |
| 16 | Were the results presented for all the analyses described in the methods? | ● |  |  |
| ***Discussion*** | | | | |
| 17 | Were the authors' discussions and conclusions justified by the results? | ● |  |  |
| 18 | Were the limitations of the study discussed? | ● |  |  |
| ***Other*** | | | | |
| 19 | Were there any funding sources or conflicts of interest that may affect the authors’ interpretation of the results? | ● |  |  |
| 20 | Was ethical approval or consent of participants attained? |  | ● | Mentioned in the study with justification |

1. ***Critcher et al 2021***

|  | **Question** | **Yes** | **No** | **Don’t know/ Comment** |
| --- | --- | --- | --- | --- |
| ***Introduction*** | | | | |
| 1 | Were the aims/objectives of the study clear? | ● |  |  |
| ***Methods*** | | | | |
| 2 | Was the study design appropriate for the stated aim(s)? | ● |  |  |
| 3 | Was the sample size justified? |  | ● |  |
| 4 | Was the target/reference population clearly defined? (Is it clear who the research was about?) | ● |  |  |
| 5 | Was the sample frame taken from an appropriate population base so that it closely represented the target/reference population under investigation? | ● |  |  |
| 6 | Was the selection process likely to select subjects/participants that were representative of the target/reference population under investigation? | ● |  |  |
| 7 | Were measures undertaken to address and categorizes non-responders? |  | ● |  |
| 8 | Were the risk factor and outcome variables measured appropriate to the aims of the study? | ● |  |  |
| 9 | Were the risk factor and outcome variables measured correctly using instruments/measurements that had been trialed, piloted or published previously? | ● |  |  |
| 10 | Is it clear what was used to determined statistical significance and/or precision estimates? (e.g. p-values, confidence intervals) | ● |  |  |
| 11 | Were the methods (including statistical methods) sufficiently described to enable them to be repeated? | ● |  |  |
| ***Results*** | | | | |
| 12 | Were the basic data adequately described? | ● |  |  |
| 13 | Does the response rate raise concerns about non-response bias? |  | ● |  |
| 14 | If appropriate, was information about non-responders described? |  | ● |  |
| 15 | Were the results internally consistent? | ● |  |  |
| 16 | Were the results presented for all the analyses described in the methods? | ● |  |  |
| ***Discussion*** | | | | |
| 17 | Were the authors' discussions and conclusions justified by the results? | ● |  |  |
| 18 | Were the limitations of the study discussed? | ● |  |  |
| ***Other*** | | | | |
| 19 | Were there any funding sources or conflicts of interest that may affect the authors’ interpretation of the results? |  | ● |  |
| 20 | Was ethical approval or consent of participants attained? |  |  | Not discussed |

1. ***Vindhyal et al 2019***

[Full text article could not be retrieved due to which abstract was analyzed]

|  | **Question** | **Yes** | **No** | **Don’t know/ Comment** |
| --- | --- | --- | --- | --- |
| ***Introduction*** | | | | |
| 1 | Were the aims/objectives of the study clear? | ● |  |  |
| ***Methods*** | | | | |
| 2 | Was the study design appropriate for the stated aim(s)? | ● |  |  |
| 3 | Was the sample size justified? |  |  | Could not deduce from the abstract |
| 4 | Was the target/reference population clearly defined? (Is it clear who the research was about?) | ● |  |  |
| 5 | Was the sample frame taken from an appropriate population base so that it closely represented the target/reference population under investigation? | ● |  |  |
| 6 | Was the selection process likely to select subjects/participants that were representative of the target/reference population under investigation? |  |  | Could not deduce from the abstract |
| 7 | Were measures undertaken to address and categorize non-responders? |  |  | Could not deduce from the abstract |
| 8 | Were the risk factor and outcome variables measured appropriate to the aims of the study? |  |  | Could not deduce from the abstract |
| 9 | Were the risk factor and outcome variables measured correctly using instruments/measurements that had been trialed, piloted or published previously? |  |  | Could not deduce from the abstract |
| 10 | Is it clear what was used to determined statistical significance and/or precision estimates? (e.g., p-values, confidence intervals) | ● |  |  |
| 11 | Were the methods (including statistical methods) sufficiently described to enable them to be repeated? |  |  | Could not deduce from the abstract |
| ***Results*** | | | | |
| 12 | Were the basic data adequately described? |  |  | Could not deduce from the abstract |
| 13 | Does the response rate raise concerns about non-response bias? |  |  | Could not deduce from the abstract |
| 14 | If appropriate, was information about non-responders described? |  |  | Could not deduce from the abstract |
| 15 | Were the results internally consistent? |  |  | Could not deduce from the abstract |
| 16 | Were the results presented for all the analyses described in the methods? | ● |  |  |
| ***Discussion*** | | | | |
| 17 | Were the authors' discussions and conclusions justified by the results? |  |  | Could not deduce from the abstract |
| 18 | Were the limitations of the study discussed? |  |  | Could not deduce from the abstract |
| ***Other*** | | | | |
| 19 | Were there any funding sources or conflicts of interest that may affect the authors’ interpretation of the results? |  |  | Could not deduce from the abstract |
| 20 | Was ethical approval or consent of participants attained? |  |  | Could not deduce from the abstract |

‘

1. ***Talal Alzahrani et al 2018***

|  | **Question** | **Yes** | **No** | **Don’t know/ Comment** |
| --- | --- | --- | --- | --- |
| ***Introduction*** | | | | |
| 1 | Were the aims/objectives of the study clear? | ● |  |  |
| ***Methods*** | | | | |
| 2 | Was the study design appropriate for the stated aim(s)? | ● |  |  |
| 3 | Was the sample size justified? |  | ● |  |
| 4 | Was the target/reference population clearly defined? (Is it clear who the research was about?) | ● |  |  |
| 5 | Was the sample frame taken from an appropriate population base so that it closely represented the target/reference population under investigation? | ● |  |  |
| 6 | Was the selection process likely to select subjects/participants that were representative of the target/reference population under investigation? | ● |  |  |
| 7 | Were measures undertaken to address and categorize non-responders? |  | ● |  |
| 8 | Were the risk factor and outcome variables measured appropriate to the aims of the study? | ● |  |  |
| 9 | Were the risk factor and outcome variables measured correctly using instruments/measurements that had been trialed, piloted or published previously? | ● |  |  |
| 10 | Is it clear what was used to determined statistical significance and/or precision estimates? (e.g. p-values, confidence intervals) | ● |  |  |
| 11 | Were the methods (including statistical methods) sufficiently described to enable them to be repeated? | ● |  |  |
| ***Results*** | | | | |
| 12 | Were the basic data adequately described? | ● |  |  |
| 13 | Does the response rate raise concerns about non-response bias? |  | ● |  |
| 14 | If appropriate, was information about non-responders described? |  | ● |  |
| 15 | Were the results internally consistent? | ● |  |  |
| 16 | Were the results presented for all the analyses described in the methods? | ● |  |  |
| ***Discussion*** | | | | |
| 17 | Were the authors' discussions and conclusions justified by the results? | ● |  |  |
| 18 | Were the limitations of the study discussed? | ● |  |  |
| ***Other*** | | | | |
| 19 | Were there any funding sources or conflicts of interest that may affect the authors’ interpretation of the results? |  |  | Not described |
| 20 | Was ethical approval or consent of participants attained? |  |  | Not described |

1. ***Falk et al 2022***

|  | **Question** | **Yes** | **No** | **Don’t know/ Comment** |
| --- | --- | --- | --- | --- |
| ***Introduction*** | | | | |
| 1 | Were the aims/objectives of the study clear? | ● |  |  |
| ***Methods*** | | | | |
| 2 | Was the study design appropriate for the stated aim(s)? | ● |  |  |
| 3 | Was the sample size justified? |  | ● |  |
| 4 | Was the target/reference population clearly defined? (Is it clear who the research was about?) | ● |  |  |
| 5 | Was the sample frame taken from an appropriate population base so that it closely represented the target/reference population under investigation? | ● |  |  |
| 6 | Was the selection process likely to select subjects/participants that were representative of the target/reference population under investigation? | ● |  |  |
| 7 | Were measures undertaken to address and categorize non-responders? |  | ● |  |
| 8 | Were the risk factor and outcome variables measured appropriate to the aims of the study? | ● |  |  |
| 9 | Were the risk factor and outcome variables measured correctly using instruments/measurements that had been trialed, piloted or published previously? | ● |  |  |
| 10 | Is it clear what was used to determined statistical significance and/or precision estimates? (e.g. p-values, confidence intervals) | ● |  |  |
| 11 | Were the methods (including statistical methods) sufficiently described to enable them to be repeated? | ● |  |  |
| ***Results*** | | | | |
| 12 | Were the basic data adequately described? | ● |  |  |
| 13 | Does the response rate raise concerns about non-response bias? |  | ● |  |
| 14 | If appropriate, was information about non-responders described? |  | ● |  |
| 15 | Were the results internally consistent? | ● |  |  |
| 16 | Were the results presented for all the analyses described in the methods? | ● |  |  |
| ***Discussion*** | | | | |
| 17 | Were the authors' discussions and conclusions justified by the results? | ● |  |  |
| 18 | Were the limitations of the study discussed? | ● |  |  |
| ***Other*** | | | | |
| 19 | Were there any funding sources or conflicts of interest that may affect the authors’ interpretation of the results? |  |  | Not discussed |
| 20 | Was ethical approval or consent of participants attained? |  | ● | Mentioned in the study with justification |

1. ***Ndunda et al***

[Full text article could not be retrieved due to which abstract was analyzed]

|  | **Question** | **Yes** | **No** | **Don’t know/ Comment** |
| --- | --- | --- | --- | --- |
| ***Introduction*** | | | | |
| 1 | Were the aims/objectives of the study clear? | ● |  |  |
| ***Methods*** | | | | |
| 2 | Was the study design appropriate for the stated aim(s)? | ● |  |  |
| 3 | Was the sample size justified? |  |  | Could not deduce from the abstract |
| 4 | Was the target/reference population clearly defined? (Is it clear who the research was about?) | ● |  |  |
| 5 | Was the sample frame taken from an appropriate population base so that it closely represented the target/reference population under investigation? | ● |  |  |
| 6 | Was the selection process likely to select subjects/participants that were representative of the target/reference population under investigation? |  |  | Could not deduce from the abstract |
| 7 | Were measures undertaken to address and categorize non-responders? |  |  | Could not deduce from the abstract |
| 8 | Were the risk factor and outcome variables measured appropriate to the aims of the study? |  |  | Could not deduce from the abstract |
| 9 | Were the risk factor and outcome variables measured correctly using instruments/measurements that had been trialed, piloted or published previously? |  |  | Could not deduce from the abstract |
| 10 | Is it clear what was used to determined statistical significance and/or precision estimates? (e.g. p-values, confidence intervals) | ● |  |  |
| 11 | Were the methods (including statistical methods) sufficiently described to enable them to be repeated? |  |  | Could not deduce from the abstract |
| ***Results*** | | | | |
| 12 | Were the basic data adequately described? |  |  | Could not deduce from the abstract |
| 13 | Does the response rate raise concerns about non-response bias? |  |  | Could not deduce from the abstract |
| 14 | If appropriate, was information about non-responders described? |  |  | Could not deduce from the abstract |
| 15 | Were the results internally consistent? |  |  | Could not deduce from the abstract |
| 16 | Were the results presented for all the analyses described in the methods? | ● |  |  |
| ***Discussion*** | | | | |
| 17 | Were the authors' discussions and conclusions justified by the results? |  |  | Could not deduce from the abstract |
| 18 | Were the limitations of the study discussed? |  |  | Could not deduce from the abstract |
| ***Other*** | | | | |
| 19 | Were there any funding sources or conflicts of interest that may affect the authors’ interpretation of the results? |  |  | Could not deduce from the abstract |
| 20 | Was ethical approval or consent of participants attained? |  |  | Could not deduce from the abstract |

1. ***Wang et al 2018***

|  | **Question** | **Yes** | **No** | **Don’t know/ Comment** |
| --- | --- | --- | --- | --- |
| ***Introduction*** | | | | |
| 1 | Were the aims/objectives of the study clear? | ● |  |  |
| ***Methods*** | | | | |
| 2 | Was the study design appropriate for the stated aim(s)? | ● |  |  |
| 3 | Was the sample size justified? |  | ● |  |
| 4 | Was the target/reference population clearly defined? (Is it clear who the research was about?) | ● |  |  |
| 5 | Was the sample frame taken from an appropriate population base so that it closely represented the target/reference population under investigation? | ● |  |  |
| 6 | Was the selection process likely to select subjects/participants that were representative of the target/reference population under investigation? | ● |  |  |
| 7 | Were measures undertaken to address and categorize non-responders? |  | ● |  |
| 8 | Were the risk factor and outcome variables measured appropriate to the aims of the study? | ● |  |  |
| 9 | Were the risk factor and outcome variables measured correctly using instruments/measurements that had been trialed, piloted or published previously? | ● |  |  |
| 10 | Is it clear what was used to determined statistical significance and/or precision estimates? (e.g. p-values, confidence intervals) | ● |  |  |
| 11 | Were the methods (including statistical methods) sufficiently described to enable them to be repeated? | ● |  |  |
| ***Results*** | | | | |
| 12 | Were the basic data adequately described? | ● |  |  |
| 13 | Does the response rate raise concerns about non-response bias? |  | ● |  |
| 14 | If appropriate, was information about non-responders described? |  | ● |  |
| 15 | Were the results internally consistent? | ● |  |  |
| 16 | Were the results presented for all the analyses described in the methods? | ● |  |  |
| ***Discussion*** | | | | |
| 17 | Were the authors' discussions and conclusions justified by the results? | ● |  |  |
| 18 | Were the limitations of the study discussed? | ● |  |  |
| ***Other*** | | | | |
| 19 | Were there any funding sources or conflicts of interest that may affect the authors’ interpretation of the results? |  | ● |  |
| 20 | Was ethical approval or consent of participants attained? | ● |  |  |

1. ***Vindhyal et al 2020***

|  | **Question** | **Yes** | **No** | **Don’t know/ Comment** |
| --- | --- | --- | --- | --- |
| ***Introduction*** | | | | |
| 1 | Were the aims/objectives of the study clear? | ● |  |  |
| ***Methods*** | | | | |
| 2 | Was the study design appropriate for the stated aim(s)? | ● |  |  |
| 3 | Was the sample size justified? |  | ● |  |
| 4 | Was the target/reference population clearly defined? (Is it clear who the research was about?) | ● |  |  |
| 5 | Was the sample frame taken from an appropriate population base so that it closely represented the target/reference population under investigation? | ● |  |  |
| 6 | Was the selection process likely to select subjects/participants that were representative of the target/reference population under investigation? | ● |  |  |
| 7 | Were measures undertaken to address and categorize non-responders? |  | ● |  |
| 8 | Were the risk factor and outcome variables measured appropriate to the aims of the study? | ● |  |  |
| 9 | Were the risk factor and outcome variables measured correctly using instruments/measurements that had been trialed, piloted or published previously? | ● |  |  |
| 10 | Is it clear what was used to determined statistical significance and/or precision estimates? (e.g. p-values, confidence intervals) | ● |  |  |
| 11 | Were the methods (including statistical methods) sufficiently described to enable them to be repeated? | ● |  |  |
| ***Results*** | | | | |
| 12 | Were the basic data adequately described? | ● |  |  |
| 13 | Does the response rate raise concerns about non-response bias? |  | ● |  |
| 14 | If appropriate, was information about non-responders described? |  | ● |  |
| 15 | Were the results internally consistent? | ● |  |  |
| 16 | Were the results presented for all the analyses described in the methods? | ● |  |  |
| ***Discussion*** | | | | |
| 17 | Were the authors' discussions and conclusions justified by the results? | ● |  |  |
| 18 | Were the limitations of the study discussed? | ● |  |  |
| ***Other*** | | | | |
| 19 | Were there any funding sources or conflicts of interest that may affect the authors’ interpretation of the results? |  |  | Not discussed |
| 20 | Was ethical approval or consent of participants attained? | ● |  |  |

1. ***Brad et al 2020***

|  | **Question** | **Yes** | **No** | **Don’t know/ Comment** |
| --- | --- | --- | --- | --- |
| ***Introduction*** | | | | |
| 1 | Were the aims/objectives of the study clear? | ● |  |  |
| ***Methods*** | | | | |
| 2 | Was the study design appropriate for the stated aim(s)? | ● |  |  |
| 3 | Was the sample size justified? | ● |  |  |
| 4 | Was the target/reference population clearly defined? (Is it clear who the research was about?) | ● |  |  |
| 5 | Was the sample frame taken from an appropriate population base so that it closely represented the target/reference population under investigation? | ● |  |  |
| 6 | Was the selection process likely to select subjects/participants that were representative of the target/reference population under investigation? | ● |  |  |
| 7 | Were measures undertaken to address and categorize non-responders? | ● |  |  |
| 8 | Were the risk factor and outcome variables measured appropriate to the aims of the study? | ● |  |  |
| 9 | Were the risk factor and outcome variables measured correctly using instruments/measurements that had been trialed, piloted or published previously? | ● |  |  |
| 10 | Is it clear what was used to determined statistical significance and/or precision estimates? (e.g. p-values, confidence intervals) | ● |  |  |
| 11 | Were the methods (including statistical methods) sufficiently described to enable them to be repeated? | ● |  |  |
| ***Results*** | | | | |
| 12 | Were the basic data adequately described? | ● |  |  |
| 13 | Does the response rate raise concerns about non-response bias? |  | ● |  |
| 14 | If appropriate, was information about non-responders described? |  | ● |  |
| 15 | Were the results internally consistent? | ● |  |  |
| 16 | Were the results presented for all the analyses described in the methods? | ● |  |  |
| ***Discussion*** | | | | |
| 17 | Were the authors' discussions and conclusions justified by the results? | ● |  |  |
| 18 | Were the limitations of the study discussed? |  | ● |  |
| ***Other*** | | | | |
| 19 | Were there any funding sources or conflicts of interest that may affect the authors’ interpretation of the results? | ● |  |  |
| 20 | Was ethical approval or consent of participants attained? |  | ● |  |

**Supplementary Figures**

Figure 1: Funnel plot for some-days users vs non-users


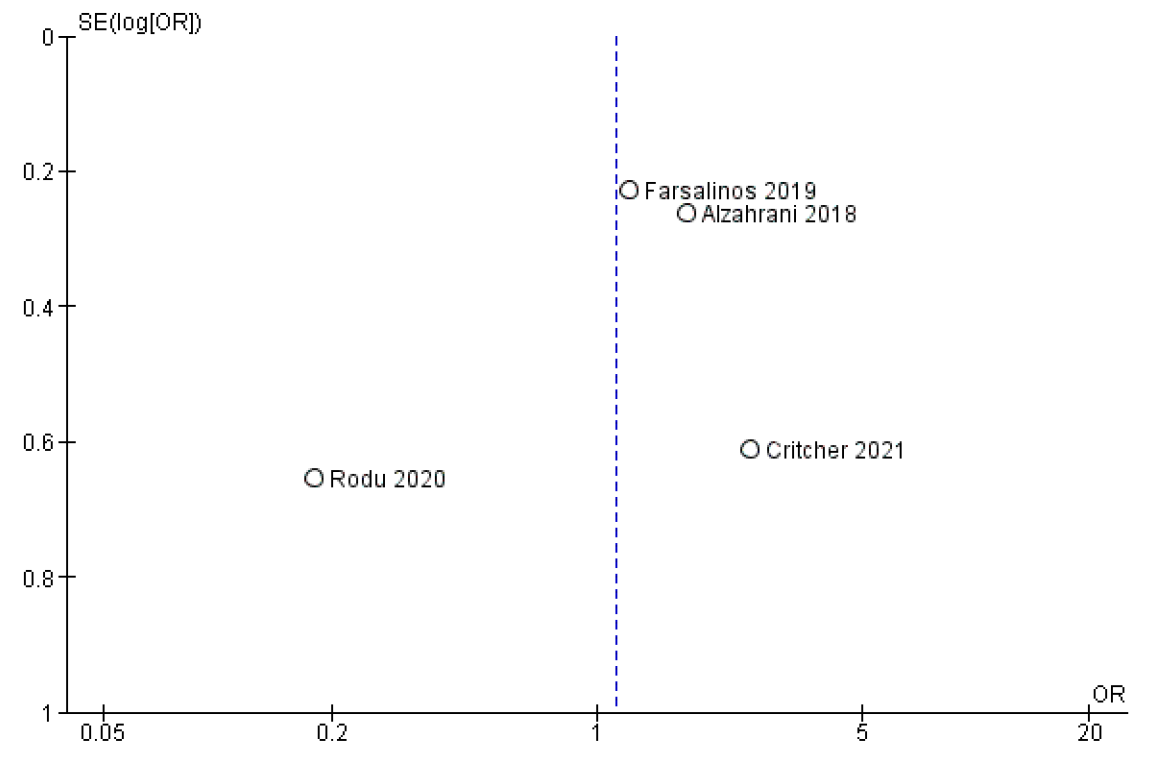

Supplement: Supplementary file 1 — Additional file 1. e-Appendix 1. AXIS quality assessment tool for observational studies. Figure 1: Funnel plot for some-days users vs non-users. [file 43044_2023_426_MOESM1_ESM.docx]
